# Cord blood adiponectin and leptin are associated with a lower risk of stunting during infancy

**DOI:** 10.1038/s41598-022-19463-3

**Published:** 2022-09-06

**Authors:** Sangshin Park, Zorimel Vargas, Anne Zhao, Palmera I. Baltazar, Jennifer F. Friedman, Emily A. McDonald

**Affiliations:** 1 Center for International Health Research, Rhode Island Hospital, Providence, RI USA; 2 Department of Pediatrics, Alpert Medical School of Brown University, Providence, RI USA; 3 Graduate School of Urban Public Health & Department of Urban Big Data Convergence, University of Seoul, 163 Seoulsiripdae-ro, Dongdaemun-gu, Seoul, 02504 Republic of Korea; 4 Brown University, Providence, RI USA; 5 Department of Immunology, Research Institute for Tropical Medicine, Manila, Philippines

**Keywords:** Biomarkers, Endocrinology

## Abstract

Undernutrition is responsible for up to 45% of deaths in children under five, with low- and middle-income countries disproportionately affected. Adipokines are known modulators of metabolism and have been linked to growth rates and neurocognition during infancy. We examined the relationship(s) between cord blood adiponectin and leptin and both longitudinal growth and cognition during the first year of life using generalized estimating equations. Infants were classified as underweight (weight-for-age z-score [WAZ]), stunted (height-for-age z-score [HAZ]) or wasted (weight-for-height z-score [WHZ]) using WHOAnthro software. Cord blood adiponectin and leptin levels were highly correlated (r = 0.35, P < 0.0001) and positively associated with birth WAZ (r = 0.34 and r = 0.45, P < 0.0001, respectively). Adipokines were independently, inversely associated with weight gain. Infants in the highest quintile of adipokine production had a lower risk of being stunted, while neither was associated with lower WAZ or WHZ in final adjusted models. Cognition was not found to be independently related to cord blood leptin or adiponectin. The negative association with adipokines and rate of weight gain during infancy may reflect heightened nutritional status at birth rather than a direct hormonal influence. The relationship between leptin or adiponectin and longitudinal length gains suggests that both adipokines may promote linear growth during infancy.

## Introduction

Stunting, or low height-for-age, is an indicator of chronic undernutrition, and poses a substantial risk to the long-term health and cognitive development of an estimated 155 million children globally, with 30% of all children under the age of five in the Philippines affected by the condition^1^. Adipose tissue is a potent endocrine organ, capable of influencing a variety of organ systems through the dynamic production of adipokines. Key among these are leptin and adiponectin, which exert opposing effects on energy metabolism; leptin being related to insulin resistance, and adiponectin an insulin sensitizer. Both are produced by the fetus, and both have been shown to be altered in cases of fetal growth restriction (FGR) in humans^2^. Many of these formerly in utero growth-restricted individuals go on to display altered response to nutrient availability after gestation^2^.

Cord blood levels of metabolic hormones have been associated with long-term growth and metabolic indicators such as pancreatic β-cell function^3–5^. Although specific mechanisms remain somewhat elusive, impacts on hypothalamic circuitry and/or epigenetic regulation have been suggested as means by which adipokines are modulated in a lasting manner in utero^6^. In addition, children who experience stunting during early childhood are at greater risk of impaired cognitive outcomes^7–10^. Fetal leptin in particular has been associated with poor neurodevelopmental outcomes, such as cognitive development in infants and hyperactivity/inattention in children^11,12^. Whether specific adipokines influence cognitive development independently, or act through undernutrition or stunting is currently unknown.

Herein, we utilized an existing cohort of infants in rural Philippines to examine the association of cord blood adiponectin and leptin with longitudinal growth during infancy and neurocognition captured by the Bayley’s Scales of Infant Development III (BSID III) at 1 year of age. These infants were recruited based on their mother’s participation in a randomized controlled trial examining the impact of praziquantel administration for the treatment of schistosomiasis during pregnancy on birth outcomes (NCT00486863).

## Materials/subjects and methods

### Ethical considerations and informed consent

This study was approved by the institutional review boards of Rhode Island Hospital and The Research Institute for Tropical Medicine (Philippines). All subjects provided informed consent prior to enrollment. All methods were performed in accordance with the relevant guidelines and regulations.

### Study site and population

Detailed recruitment and characteristics of this study population have been described elsewhere^13^. Briefly, women were recruited from 72 barangays in rural northeastern Leyte, the Philippines. This region is endemic for schistosomiasis and soil-transmitted helminths, but not malaria. HIV prevalence in the area was under 1%. Women were eligible to participate in the original study if they were over 18 years of age, otherwise healthy as determined by medical history, physical examination and laboratory studies (renal and liver function tests, complete blood count), and carrying a singleton pregnancy free from congenital defects as determined by ultrasound between 12 and 16 weeks of gestation. All women were positive for schistosomiasis and were randomized to praziquantel treatment to resolve infection, or placebo at 14 ± 2 weeks gestation. Of note, treatment did not impact birth weight, risk for low birth weight (LBW), small for gestational age (SGA), or neurodevelopmental outcomes at 12 months of age^13,14^. Women were enrolled (n = 346) at 14 ± 2 weeks of gestation, and characteristics including socioeconomic status, age and current alcohol consumption were obtained. BMI was calculated based on weight and height measurements taken at enrollment, with women with a BMI of > 25 classified as overweight. All women in this cohort delivered vaginally and infants were followed until 12 months of age.

### Sample collection

At maternal enrollment, we collected health related epidemiologic and demographic data, described in our original publication^13^. Geohelminth (*A. lumbricoides*, hookworm, *T. trichuria*) and schistosome infections were determined at enrollment and again approximately 8 weeks after treatment. Cord blood was collected directly from the umbilical cord after delivery of the placenta and cleaning of the outside of the umbilical cord with alcohol wipe(s). Blood samples were collected in a purple top vacutainer tubes and plasma obtained after centrifuging for 15 min at 5000×*g*.

### Anthropometric measurements

Newborn weight and length were measured within 24 h of delivery, and height, weight and mid-upper arm circumference (MUAC) and head circumference collected at 1 month, 6 months and 1 year of age. Breast feeding status (exclusive, mixed, none) was collected via questionnaires administered to the mother at each visit. Low birth weight was classified as infants weighing < 2500×*g* at birth. Infants were classified as preterm if delivery was prior to 37 weeks gestation, as determined by ultrasound at enrollment. Small-for-gestational age (SGA) was calculated using the application developed by the INTERGROWTH-21th project^15^. Height-for-age z-scores (HAZ), weight-for-age z-score (WAZ), weight-for-height z-scores (WHZ) and MUAC z-scores were calculated using the 2006 World Health Organization Anthro Growth Standards (www.who.int/childgrowth/software/en/). Infants with individual z-scores below two standard deviations of the mean were considered stunted (HAZ), underweight (WAZ) or wasted (WHZ) during infancy.

### Adipokine measurement

Total adiponectin levels in cord blood plasma were measured using commercially available ELISA kits (R&D Systems, Minneapolis, MN). Intra assay %CV for adiponectin was 5.54%, inter assay %CV was 4.03%. Leptin was analyzed by generation of a bead-based Luminex system (BioRad, Hercules, CA), with the lower limit of detection being 7.8 pg/mL. Intra assay %CV for leptin was 4.41%, inter assay %CV was 8.2%.

### Cognition assessments

Cognition was assessed at 12 months using the Bayley Scales of Infant and Toddler Development test, third edition (BSID-III) by trained study pediatricians^16^. This test consists of three primary developmental categories: cognitive, language (consisting of receptive and expressive language subscales), and motor (consisting of fine and gross motor subscales). As reported previously, the social scales were not included in this examination^14^. Cognition, receptive language, expressive language, fine motor and gross motor skills were scaled according to age grouping by month and examined for relationship(s) with either cord blood adiponectin or leptin.

### Statistical analysis

Statistical analysis was carried out using SAS and generalized estimated equations (GEE) using a Poisson distribution with log link, an unstructured correlation structure, and robust variance estimation^17^. All models included SES and breastfeeding status. A stepwise selection approach was used for each model selection. We considered predictors with *P* < 0.2 in univariable analysis for inclusion in multivariable analysis. We included predictors with *P* < 0.05 in the multivariable models in the presence of adiponectin or leptin variables. *P* < 0.05 was considered as significant.

## Results

General characteristics of the study population are presented in Table 1. Adiponectin levels in cord blood were not different based on maternal factors including first trimester BMI, socioeconomic status (SES), age, or level of education. Females did show higher levels of adiponectin than male infants (*P* = 0.05). The highest levels of adiponectin were also statistically associated with breastfeeding practices (*P* = 0.024); we therefore included breastfeeding status in all subsequent statistical modeling. Cord blood leptin levels were not associated with maternal SES, age, level of education or alcohol consumption during pregnancy. Cord blood leptin levels were positively associated with maternal BMI. Similar to what was observed with adiponectin, cord blood leptin levels were higher in female neonates.

**Table 1:** General characteristics of the infant cohort.

Variable	n	Quintiles of adipokines in cord blood					*P* for trend
		Adiponectin					
		Q1	Q2	Q3	Q4	Q5	
Range, µg/mL		0.4 to 7.6	7.7 to 11.8	12.0 to 16.3	16.5 to 21.1	21.1 to 36.7	
n		64	66	66	65	64	
**Maternal factor**							
Age (≥ 30 years), %	325	26.6	37.9	39.4	32.3	26.6	0.76
Overweight, %	325	9.4	21.2	24.2	21.5	15.6	0.41
High SES (≥ median), %	325	46.9	54.6	47.0	46.2	56.3	0.61
Higher education (≥ high school), %	323	60.9	65.2	52.3	50.0	57.8	0.27
Alcohol consumption, %	325	82.8	69.7	86.4	76.9	73.4	0.49
Gestational weight gain, kg	323	5.73 ± 2.04	5.64 ± 2.25	5.93 ± 2.37	5.53 ± 2.47	6.49 ± 2.21	0.12
**Infant factor**							
Female, %	325	37.5	47.0	40.9	47.7	56.3	0.05
Premature, %	325	17.2	9.1	4.6	3.1	10.9	0.10
Breastfeeding^a^, %	282	82.8	82.5	90.2	94.8	91.7	0.024
At birth							
LBW, %^b^	325	23.4	19.7	13.6	6.2	6.3	< 0.001
SGA, %^b^	325	31.3	31.8	27.3	16.9	10.9	< 0.001
WAZ	325	− 1.47 ± 1.27	− 1.11 ± 0.84	− 0.94 ± 0.92	− 0.80 ± 0.76	− 0.63 ± 0.82	< 0.001
HAZ	325	− 1.61 ± 1.73	− 1.70 ± 1.61	− 1.33 ± 1.68	− 1.03 ± 1.50	− 1.50 ± 2.40	0.20
WHZ	254	− 0.55 ± 1.77	− 0.56 ± 1.48	− 0.35 ± 1.18	− 0.34 ± 1.75	− 0.50 ± 1.84	0.64
Apgar in 1 min	323	7.33 ± 1.63	7.89 ± 1.12	8.20 ± 1.00	7.89 ± 1.16	8.34 ± 1.32	< 0.001
Apgar in 5 min	316	8.23 ± 1.26	8.81 ± 0.94	8.98 ± 0.86	8.81 ± 0.95	9.08 ± 0.90	< 0.001
At 12 mo							
WAZ	292	− 1.59 ± 0.97	− 1.74 ± 1.12	− 1.57 ± 0.93	− 1.74 ± 0.97	− 1.42 ± 0.86	0.48
HAZ	292	− 1.24 ± 1.11	− 1.25 ± 1.06	− 1.35 ± 0.98	− 1.21 ± 0.95	− 1.05 ± 0.92	0.35
WHZ	292	− 1.33 ± 1.04	− 1.54 ± 1.18	− 1.23 ± 0.99	− 1.57 ± 1.00	− 1.25 ± 0.87	0.83
**BSID-III**							
Cognitive	292	10.71 ± 2.37	10.77 ± 1.67	11.02 ± 1.95	10.54 ± 2.47	10.85 ± 2.15	0.96
Receptive language	292	7.53 ± 1.54	7.53 ± 2.64	7.27 ± 1.72	6.98 ± 1.14	6.91 ± 1.14	0.014
Expressive language	292	7.19 ± 2.19	7.12 ± 1.8	6.92 ± 1.7	7.1 ± 1.26	6.65 ± 1.25	0.13
Fine motor	292	10.63 ± 2.55	9.84 ± 2.17	10.13 ± 2.16	9.32 ± 2.32	9.84 ± 2.19	0.025
Gross motor	292	8.95 ± 3.74	8.74 ± 2.93	9 ± 2.82	8.97 ± 2.41	9.76 ± 2.71	0.14

Variable		Leptin					
Range, ng/mL		0.1 to 2.3	2.4 to 4.7	4.8 to 7.0	7.0 to 13.3	13.3 to 75.1	
N		68	67	70	68	68	
**Maternal factor**							
Age (≥ 30 y), %	341	35.3	28.4	38.6	33.8	29.4	0.72
Overweight, %	341	8.8	13.4	18.6	26.5	19.1	0.021
High SES (≥ median), %	341	48.5	50.8	41.4	48.5	60.3	0.27
Higher education (≥ high school), %	339	53.7	64.2	59.4	55.9	57.4	0.95
Alcohol consumption, %	341	80.9	74.6	75.7	79.4	70.6	0.33
Gestational weight gain, kg	340	5.49 ± 2.06	6.03 ± 2.06	5.73 ± 2.30	6.12 ± 2.42	6.39 ± 2.64	0.032
**Infant factor**							
Female, %	341	27.9	40.3	42.9	57.4	64.7	< 0.001
Premature, %	341	11.8	14.9	4.3	8.8	5.9	0.11
Breastfeeding^a^, %	289	85.3	84.2	91.9	89.8	90.0	0.26
At birth							
LBW, %^b^	341	33.8	19.4	8.6	1.5	5.9	< 0.001
SGA, %^b^	341	50.0	26.9	22.9	5.9	8.8	< 0.001
WAZ	341	− 1.67 ± 1.05	− 1.21 ± 0.87	− 0.93 ± 0.70	− 0.69 ± 0.63	− 0.44 ± 0.83	< 0.001
HAZ	341	− 1.67 ± 1.55	− 1.61 ± 1.90	− 1.38 ± 1.64	− 1.17 ± 1.84	− 1.25 ± 2.08	0.06
WHZ	269	− 1.11 ± 1.83	− 0.82 ± 1.57	− 0.31 ± 1.69	− 0.32 ± 1.27	0.14 ± 1.37	< 0.001
Apgar in 1 min	339	7.71 ± 1.58	8.09 ± 1.10	7.93 ± 1.20	8.03 ± 1.18	8.12 ± 1.17	0.11
Apgar in 5 min	332	8.56 ± 1.19	8.72 ± 0.90	8.82 ± 0.96	8.85 ± 0.88	9.06 ± 0.90	0.003
At 12 mo							
WAZ	299	− 1.78 ± 0.96	− 1.70 ± 1.13	− 1.61 ± 0.87	− 1.60 ± 0.96	− 1.39 ± 0.85	0.029
HAZ	299	− 1.52 ± 1.04	− 1.24 ± 1.18	− 1.20 ± 0.86	− 1.09 ± 0.83	− 1.06 ± 0.92	0.007
WHZ	299	− 1.40 ± 1.07	− 1.49 ± 1.07	− 1.40 ± 0.96	− 1.46 ± 0.99	− 1.20 ± 0.96	0.34
**BSID-III**							
Cognitive	299	10.68 ± 2.14	11.16 ± 2.03	10.56 ± 2.02	10.31 ± 1.98	11.21 ± 2.44	0.90
Receptive language	299	7.35 ± 1.76	7.39 ± 1.91	7.19 ± 1.54	7.00 ± 1.89	7.13 ± 1.40	0.23
Expressive language	299	6.85 ± 1.63	7.29 ± 2.11	7.13 ± 1.24	6.66 ± 1.70	6.92 ± 1.52	0.48
Fine motor	299	10.08 ± 2.29	10.08 ± 2.41	9.89 ± 2.27	9.66 ± 2.20	9.91 ± 2.41	0.40
Gross motor	299	8.98 ± 2.96	8.84 ± 3.43	9.27 ± 2.38	8.85 ± 2.99	9.28 ± 2.72	0.63

*SES* socioeconomic status, *LBW* low birth weight, *SGA* small for gestational age, *WAZ* weight-for-age z-score, *HAZ* height-for-age z-score, *WHZ* weight-for-height z-score, *BSID-III* the Bayley Scales of Infant and Toddler Development test, third edition.

^a^Percent of infants reported to be exclusively breastfed throughout the first year of life.

^b^Percent of infants classified as LBW or SGA at birth.

### Association of cord blood adipokines with growth during infancy

Cord blood levels of leptin and adiponectin were highly correlated with one another, regardless of maternal parameters (Table 2). Both cord plasma adiponectin and leptin were independently correlated with weight for gestational age at delivery, as captured by SGA (Table 1), with both adipokines higher in heavier newborns. Additionally, there was a trend for a positive association between cord blood leptin and length at delivery (Table 1). Cord blood leptin levels were also positively correlated with weight-for-age (WAZ) and height-for-age (HAZ) at 1 year (Tables 1 and 2). In contrast, leptin and adiponectin in cord blood were both negatively correlated with the change in WAZ over the first year of life in this cohort (Table 2). Similarly, cord blood leptin was negatively correlated with the change in WHZ over the first year of infancy (Table 2).

**Table 2:** Pearson correlation coefficients between cord blood adipokines and infant growth.

Variable	2.	3.	4.	5.	6.	7.	8.	9.	10.	11.
1. Adiponectin in cord blood	0.35***	0.35***	0.09	0.04	0.05	0.1	− 0.01	− 0.27***	− 0.04	− 0.06
2. Leptin in cord blood		0.45***	0.10	0.25***	0.13*	0.15**	0.06	− 0.32***	− 0.03	− 0.19**
3. WAZ at birth			0.32***	0.53***	0.36***	0.38***	0.21***	− 0.56***	− 0.11	− 0.36***
4. HAZ at birth				− 0.59***	0.22***	0.25***	0.12*	− 0.09	− 0.84***	0.57***
5. WHZ at birth					0.09	− 0.03	0.15*	− 0.34***	0.52***	− 0.83***
6. WAZ at 12 mo						0.67***	0.85***	0.57***	0.15**	0.41***
7. HAZ at 12 mo							0.2***	0.27***	0.31***	0.15*
8. WHZ at 12 mo								0.57***	− 0.01	0.44***
9. WAZ gain/mo									0.23***	0.66***
10. HAZ gain/mo										− 0.40***
11. WHZ gain/mo										

Adiponectin (μg/mL) and leptin (ng/mL) were ln-transformed.

*WAZ* weight-for-age z-score, *HAZ* height-for-age z-score, *WHZ* weight-for-height z-score.

*P value < 0.05, **P value < 0.01, ***P value < 0.001.

**Figure 1:**
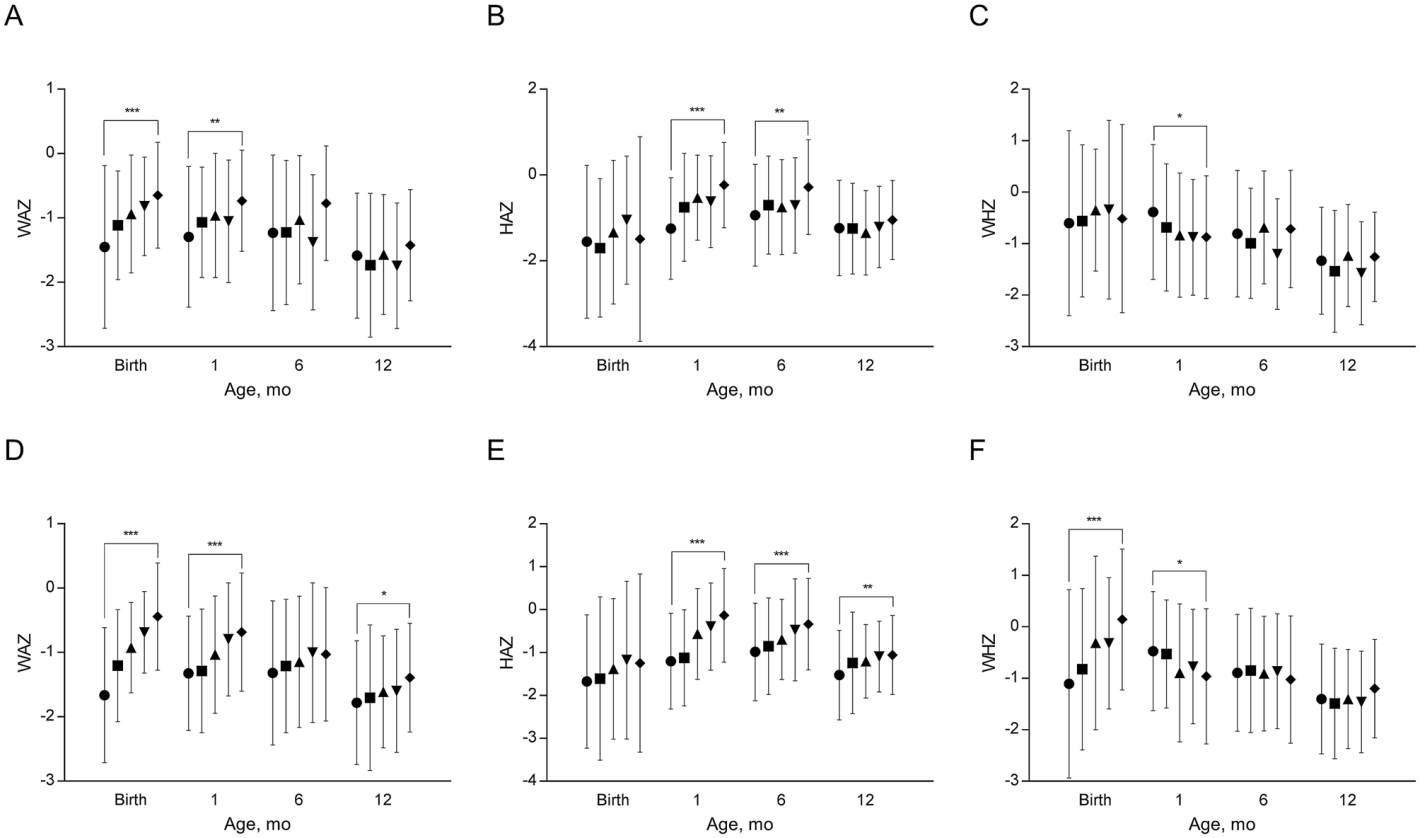
Weight-for-age z-score (WAZ), height-for-age z-score (HAZ), and weight-for-height z-score (WHZ) according to quintiles of adiponectin (**A–C**) and leptin (**D–F**) in cord blood. Error bar indicates standard deviation. Filled circle: quintile 1—the lowest level, filled square: quintile 2, filled triangle: quintile 3, filled inverted triangle: quintile 4, filled diamond: quintile 5—the highest level. *P value < 0.05, **P value < 0.01, ***P value < 0.001.

In univariate analysis, cord blood adiponectin was positively associated with weight-for-age z-score (WAZ) early in infancy (delivery and 1 month; Fig. 1A), positively associated with height-for-age z-score (HAZ) at 1 and 6 months (Fig. 1B), but negatively associated with weight-for-height z-score (WHZ) at 1 month (Fig. 1C). All associations between cord blood adiponectin and infant growth parameters were lost by 12 months of age. Similarly, cord blood leptin levels were positively associated with HAZ at 1 and 6 months of age (Fig. 1E) and negatively with WHZ at 1 month of age (Fig. 1F). The positive association between cord blood leptin and WAZ however was more persistent than that observed with adiponectin, lasting through the first year of infancy (Fig. 1D).

### Adipokines as predictors of growth during infancy

Univariate analysis of the relationship between cord blood adiponectin and leptin and longitudinal growth are presented in Supplemental Table 1. We have previously reported the association of maternal and infant characteristics with poor growth outcomes during infancy in this population^18^, and used these data to inform our multivariable analysis of the relationship between cord blood adiponectin and leptin and growth during infancy (Table 3). We found the highest level of adiponectin levels in cord blood to be negatively associated with risk of stunting during infancy (RR: 0.32 (0.16–0.64)). Similarly, elevated leptin levels in cord blood were negatively associated with risk of stunting in infancy (RR: 0.39 (0.18–0.83) and RR: 0.36 (0.17–0.76) for the two highest quintiles). These associations remained when adiponectin and leptin were analyzed as continuous variables (Table 4). Neither adipokine was associated with risk of developing underweight or wasting during infancy.

**Table 3:** Multivariable generalized estimated equation models of adiponectin and leptin quintiles predicting underweight, stunting, and wasting during infancy.

Outcome (covariates)	Quintiles of adipokines in cord blood					*P* for trend
	Predictor: adiponectin					
	Q1	Q2	Q3	Q4	Q5	
Range, µg/mL	0.4 to 7.6	7.7 to 11.8	12.0 to 16.3	16.5 to 21.1	21.1 to 36.7	
n	64	66	66	65	64	
Underweight^a^	Ref	1.35 (0.87–2.08)	1.11 (0.69–1.81)	1.58 (0.95–2.61)	0.91 (0.47–1.73)	0.78
Stunting^b^	Ref	0.92 (0.54–1.56)	0.88 (0.52–1.51)	0.81 (0.42–1.57)	0.32 (0.16–0.64)**	0.004
Wasting^c^	Ref	1.27 (0.78–2.06)	0.93 (0.54–1.60)	1.43 (0.89–2.29)	1.10 (0.64–1.88)	0.56

Outcome (covariates)	Predictor: leptin					
Range, ng/mL	0.1 to 2.3	2.4 to 4.7	4.8 to 7.0	7.0 to 13.3	13.3 to 75.1	
n	68	67	70	68	68	
Underweight^a^	Ref	1.33 (0.88–2.01)	1.11 (0.71–1.76)	1.21 (0.73–2.00)	1.45 (0.85–2.47)	0.30
Stunting^b^	Ref	0.88 (0.57–1.36)	0.56 (0.31–1.00)	0.39 (0.18–0.83)*	0.36 (0.17–0.76)**	< 0.001
Wasting^c^	Ref	1.15 (0.71–1.85)	1.14 (0.71–1.85)	1.07 (0.66–1.72)	1.17 (0.70–1.93)	0.68

^a^Adjusted for maternal age, socioeconomic status (SES), sex, and weight-for-age z-score at birth.

^b^Adjusted for SES, premature, exclusively breastfeeding, and height-for-age z-score at birth.

^c^Adjusted for maternal age and SES.

**Table 4:** Multivariable generalized estimated equation models of continuous adiponectin and leptin levels predicting underweight, stunting, and wasting during infancy.

Outcome (covariates)	RR (95% CI)	*P* value
**Predictor: adiponectin in cord blood, µg/mL**		
Underweight^a^	1.09 (0.88–1.34)	0.43
Stunting^b^	0.81 (0.66–0.99)	0.037
Wasting^c^	1.03 (0.80–1.31)	0.83
**Predictor: leptin in cord blood, ng/mL**		
Underweight^a^	1.05 (0.91–1.22)	0.50
Stunting^b^	0.72 (0.61–0.84)	< 0.001
Wasting^c^	1.05 (0.89–1.23)	0.57

^a^Adjusted for maternal age, socioeconomic status (SES), sex, and weight-for-age z-score at birth.

^b^Adjusted for SES, exclusively breastfeeding, and height-for-age z-score at birth.

^c^Adjusted for maternal age and SES.

### Associations with cognitive outcomes

In univariate analysis, the highest quintile of cord blood adiponectin was negatively associated with receptive language and fine motor scores, while not significantly associated with cognition, expressive language or gross motor skills scores. Cord blood leptin was not associated with any of the cognitive scales examined in univariate analysis (Table 1). We have previously reported on predictors of cognition in this population, such as maternal iron status, infant growth parameters, maternal education and cognition^14^. Multivariable regression models with specific predictors for a given cognitive outcome included failed to show an association between either adiponectin or leptin and most of the cognitive outcomes assessed. The exception was the negative association between cord blood adiponectin and fine motor skills was retained after inclusion of maternal cognition and education (β: − 0.058, *P* = 0.004), two factors we previously showed to be associated with fine motor skill development in this population (data not shown)^14^.

## Discussion

Herein, we have examined the association of fetal adiponectin and leptin with growth during the first year of life. Since its inception^19^, the Developmental Origins of Health and Disease hypothesis has been substantiated by a wealth of studies, and the role of the metabolic axis as established during gestation on subsequent growth in infancy is substantial. Infancy, in turn, represents a critical period of growth and development, both because of the heightened trajectory by which both of these processes occur during this period, and also due to the known associations between growth in infancy and long-term impacts on the affected individual, including cognition, longitudinal growth potential, and establishment of risk for metabolic diseases late in life. As such, identification of biomarker(s) which may predict growth trajectory during infancy may serve to identify those infants most at risk of poor growth characteristics in time for an appropriate intervention to be made.

Infancy and early childhood is the most likely time period during which stunting will occur, largely due to this being the period of life with the greatest growth velocity, poor quality of weaning foods, multiple infectious disease insults that can affect appetite and gut health, and the need for energy dense foods^20^. The importance of growth during the period of infancy on adult attained height and adiposity has been demonstrated by others^21^. Growth trajectory during the first 2 years of life has been associated with adult size, independent of BMI at birth or 2 years, highlighting that the *change* in growth during this period as critical to determining adult size^21^. In addition, children born SGA who failed to display catch up growth have reduced adiponectin and insulin sensitivity in childhood, compared to height matched children born AGA^22^. Levels of adiponectin in cord blood are also positively associated with birth weight, being lower in small-for-gestational-age (SGA) infants in some, but not all studies^23–29^. Our study substantiates these findings, showing significantly more low birth weight infants falling into the lowest quintiles for cord blood adiponectin.

Studies examining the association with cord blood adiponectin and growth and nutritional status during infancy have reported mixed results, with overall consensus suggesting a slight positive association with cord adiponectin and neonatal adiposity^30^. Cord blood adiponectin has also been negatively associated with weight gain during early infancy^31^, and weight-for-age in the first year of life^32^, however others have reported a positive association or none at all^5,29,33,34^. Indeed, a recent report suggests cord blood adiponectin is predictive of central adiposity at 3 years of age^35^. While we observed a significant association with the highest levels of cord blood adiponectin and weight-for-age z-scores, these effects were limited to only the first month of life, and overall cord adiponectin was actually negatively associated with weight-for-age gain during infancy. This suggests that adiponectin’s relationship with weight for age is driven by its effects on size at birth. In addition, the relationship between cord blood adiponectin and growth trajectories may display sex discordance^36,37^. Our data also substantiate these findings, with elevated adiponectin in female neonates and disparate rates of growth between the sexes^18^.

Adiponectin has also been associated with long bone growth, and evidence in mouse models show it plays a role in promoting osteogenesis^38^. These data are in line with our observation of higher cord blood adiponectin being related to decreased risk of stunting (or to higher HAZ) during infancy, but not underweight or wasting. Interestingly, others have reported an association with neonatal adiponectin and wasting, but not stunting^39^. This study examined intrauterine stunting as a subtype of SGA, therefore our definition of stunting based on growth during infancy could help explain our divergent results, as well as potential divergent genetic and environmental responses based on the unique study populations.

Cord blood leptin is known to be positively associated with birthweight^28,40,41^. Cord blood leptin has also been reported to exhibit a positive association with growth trajectory during infancy into early childhood in infants with normal growth patterns. In contrast, leptin has been inversely associated with both catchup growth during early infancy and, among SGA infants, with catchup growth, highlighting the importance of leptin in mediating the metabolic axis^35,42^. This is particularly important in a resource limited environment, and highlights the importance of understanding primary determinants of growth during infancy. We observed a positive association between cord blood leptin and weight-for-age at delivery, as well weight-for-age at 1 year of age. While we did not dichotomize our data based on SGA status, cord blood leptin was negatively associated with the weight-for-age gain/month during infancy, suggesting that our study may be in accordance with reports demonstrating a negative association between cord leptin and rates of catchup growth during infancy.

Cognitive development during infancy is known to be highly influenced by inflammatory events during this period, while growth is predominantly associated with maternal body size and birth anthropometry^43^. Adiponectin is known to exert anti-inflammatory effects, and in an aged model, adiponectin has been shown to improve cognitive performance through suppression of the inflammatory cascade, in addition to its impacts on appetite regulation and energy expenditure^44^. Similarly, cord blood leptin has been associated with a reduced risk of hyperactivity problems in school-aged children^11^. However plasma leptin in infants has also been reported to be negatively related to cognitive outcomes using the BSID-III test^12^. While we observed a slight negative association between the highest levels of cord blood adiponectin and receptive language and fine motor scores at 12 months of age, only the relationship between cord adiponectin and fine motor skills was maintained after adjustment for key confounders (i.e. maternal cognition score and education). We did not observe any association between cord blood leptin and any of the cognitive outcomes measured. These data suggest that while there may be a slight negative association between cord blood adiponectin and some cognitive development parameters, larger impacts for either adipokine, as reported by others, may be mediated indirectly through other pathways, such as growth and nutritional status.

Our study is limited by measurement of adiponectin and leptin only at delivery, and future studies examining the relationship of adiponectin and leptin and growth throughout infancy will enhance our understanding of the dynamic role these hormones play in mediating the metabolic axis during infancy. In addition, our study represents a secondary analysis from a cohort of infants, all of whom were born to mothers with schistosomiasis early in gestation. Half of these mothers received praziquantel for resolution of infection early in gestation, and treatment did not impact birth weight of the neonates^13^, such that we do not expect maternal treatment impacted our findings. Similarly, treatment group was not associated with cord blood adipokine levels. Our study was also limited to women residing in rural villages within the Philippines, potentially limiting the applicability of our findings to other ethnicities/regions. In addition, we measured total adiponectin, rather than focusing exclusively on the high molecular weight (HMW) form of adiponectin which is known to be the most bioactive form of adiponectin.

In conclusion, we failed to identify an association with either leptin or adiponectin cord blood levels and change in adiposity during the first year of infancy. Tracking of these adipokines throughout infancy as they relate to growth may help identify more nuanced associations throughout this highly dynamic period, and is an area of continued interest for our group. We did however observe a positive association with both cord blood adiponectin and leptin and *gain* in length during the first year. This association remained after controlling for length at delivery, suggesting that these adipokines may be independently associated with long bone growth during infancy, rather than simply a reflection of birth length or continued growth by a large neonate.

## Supplementary Information

Supplementary Table 1.

## Data Availability

The datasets generated and/or analysed during the current study are not publicly available because the dataset contains confidential patient data but are available from the corresponding author on reasonable request.
