# Supplementary material for: Cord blood adiponectin and leptin are associated with a lower risk of stunting during infancy
Source: Sci Rep. 2022 Sep 6;12:15122. doi: 10.1038/s41598-022-19463-3 (PMC9448758; doi:10.1038/s41598-022-19463-3)
Supplement: Supplementary file 1 — Supplementary Table 1. [file 41598_2022_19463_MOESM1_ESM.docx]

**Supplemental Table 1. Univariable generalized estimated equation models predicting underweight, stunting, and wasting during infancy.**

|  | Underweight | | Stunting | | Wasting | |
| --- | --- | --- | --- | --- | --- | --- |
|  | RR (95% CI) | *P* value | RR (95% CI) | *P* value | RR (95% CI) | *P* value |
| Maternal factor |  |  |  |  |  |  |
| Age (≥30 y), % | 1.55 (1.14-2.11) | 0.005 | 1.09 (0.72-1.66) | 0.69 | 1.59 (1.18-2.15) | 0.002 |
| Overweight, % | 1.04 (0.70-1.55) | 0.85 | 1.12 (0.66-1.89) | 0.68 | 0.97 (0.63-1.50) | 0.89 |
| High SES (≥median), % | 0.66 (0.48-0.90) | 0.009 | 0.59 (0.40-0.89) | 0.011 | 0.74 (0.54-1.00) | 0.048 |
| Higher education (≥high school), % | 0.78 (0.57-1.06) | 0.11 | 0.86 (0.58-1.27) | 0.46 | 0.72 (0.53-0.98) | 0.037 |
| Alcohol consumption, % | 0.97 (0.67-1.41) | 0.89 | 1.38 (0.82-2.34) | 0.22 | 0.86 (0.62-1.20) | 0.39 |
| Infant factor |  |  |  |  |  |  |
| Female, % | 0.59 (0.43-0.82) | 0.002 | 0.60 (0.39-0.92) | 0.018 | 0.85 (0.63-1.15) | 0.30 |
| Premature, % | 2.22 (1.54-3.20) | <0.001 | 2.78 (1.78-4.33) | <0.001 | 1.13 (0.70-1.81) | 0.63 |
| Breastfeeding, % | 0.73 (0.47-1.14) | 0.17 | 0.48 (0.30-0.79) | 0.004 | 1.52 (0.84-2.75) | 0.16 |
| At birth |  |  |  |  |  |  |
| WAZ | 0.59 (0.54-0.64) | <0.001 | 0.55 (0.50-0.62) | <0.001 | 0.80 (0.70-0.91) | 0.001 |
| HAZ | 0.85 (0.79-0.91) | <0.001 | 0.82 (0.75-0.89) | <0.001 | 0.95 (0.88-1.02) | 0.14 |
| WHZ | 0.89 (0.81-0.98) | 0.020 | 1.01 (0.87-1.17) | 0.89 | 0.94 (0.86-1.03) | 0.20 |
| BSID-III |  |  |  |  |  |  |
| Cognitive | 0.95 (0.88-1.02) | 0.13 | 0.99 (0.92-1.07) | 0.85 | 0.93 (0.86-1.00) | 0.047 |
| Receptive language | 0.91 (0.80-1.04) | 0.15 | 1.06 (0.95-1.18) | 0.33 | 0.84 (0.74-0.95) | 0.005 |
| Expressive language | 0.88 (0.80-0.98) | 0.019 | 0.96 (0.85-1.08) | 0.50 | 0.93 (0.84-1.03) | 0.17 |
| Fine motor | 0.99 (0.93-1.06) | 0.85 | 1.03 (0.95-1.12) | 0.51 | 0.96 (0.90-1.03) | 0.26 |
| Gross motor | 0.94 (0.89-1.00) | 0.042 | 1.00 (0.95-1.06) | 0.89 | 0.95 (0.89-1.00) | 0.07 |
| Adiponectin in cord blood |  |  |  |  |  |  |
| Q1 (0.4 to 7.6 µg/mL) | Ref |  | Ref |  | Ref |  |
| Q2 (7.7 to 11.8 µg/mL) | 0.93 (0.58-1.47) | 0.75 | 0.82 (0.48-1.42) | 0.48 | 1.30 (0.80-2.09) | 0.29 |
| Q3 (12.0 to 16.3 µg/mL) | 0.75 (0.45-1.24) | 0.26 | 0.71 (0.41-1.23) | 0.22 | 1.00 (0.58-1.72) | 0.99 |
| Q4 (16.5 to 21.1 µg/mL) | 0.93 (0.59-1.48) | 0.76 | 0.61 (0.31-1.17) | 0.13 | 1.50 (0.93-2.41) | 0.10 |
| Q5 (21.1 to 36.7µg/mL) | 0.44 (0.23-0.83) | 0.012 | 0.30 (0.15-0.58) | <0.001 | 1.05 (0.61-1.79) | 0.87 |
| P for trend | 0.019 |  | <0.001 |  | 0.65 |  |
| Leptin in cord blood |  |  |  |  |  |  |
| Q1 (0.1 to 2.3 ng/mL) | Ref |  | Ref |  | Ref |  |
| Q2 (2.4 to 4.7 ng/mL) | 0.82 (0.53-1.27) | 0.38 | 0.91 (0.58-1.42) | 0.67 | 1.11 (0.68-1.80) | 0.68 |
| Q3 (4.8 to 7.0 ng/mL) | 0.63 (0.39-1.02) | 0.06 | 0.50 (0.27-0.92) | 0.026 | 1.19 (0.73-1.95) | 0.49 |
| Q4 (7.0 to 13.3 ng/mL) | 0.53 (0.32-0.86) | 0.010 | 0.35 (0.17-0.72) | 0.005 | 1.06 (0.65-1.72) | 0.82 |
| Q5 (13.3 to 75.1 ng/mL) | 0.51 (0.30-0.87) | 0.013 | 0.34 (0.17-0.67) | 0.002 | 1.07 (0.64-1.78) | 0.81 |
| P for trend | 0.002 |  | <0.001 |  | 0.89 |  |
| Continuous adiponectin in cord blood, µg/mL | 0.71 (0.58-0.86) | <0.001 | 0.67 (0.53-0.84) | <0.001 | 1.02 (0.80-1.29) | 0.90 |
| Continuous leptin in cord blood, ng/mL | 0.77 (0.67-0.89) | <0.001 | 0.70 (0.59-0.82) | <0.001 | 1.03 (0.88-1.20) | 0.72 |

WAZ: weight-for-age z-score, HAZ: height-for-age z-score, WHZ: weight-for-height z-score. Continuous adiponectin (μg/ml) and leptin (ng/ml) were ln-transformed.
